# Preclinical Considerations about Affective Disorders and Pain: A Broadly Intertwined, yet Often Under-Explored, Relationship Having Major Clinical Implications

**DOI:** 10.3390/medicina56100504

**Published:** 2020-09-25

**Authors:** Iulia Antioch, Ovidiu-Dumitru Ilie, Alin Ciobica, Bogdan Doroftei, Michele Fornaro

**Affiliations:** 1 Department of Research, Faculty of Biology, “Alexandru Ioan Cuza” University, Carol I Avenue, no 11, 700505 Iasi, Romania; iulia.antioch@gmail.com (I.A.); ovidiuilie90@yahoo.com (O.-D.I.); 2 Faculty of Medicine, University of Medicine and Pharmacy “Grigore T. Popa”, University Street, no 16, 700115 Iasi, Romania; 3 Department of Psychiatry, New York State Psychiatric Institute, Columbia University, New York, NY 10027, USA; dott.fornaro@gmail.com

**Keywords:** pain, major depressive disorder, bipolar disorder, gastrointestinal

## Abstract

*Background:* Pain, a distinctive undesirable experience, encompasses several different and fluctuating presentations across varying mood disorders. Therefore, the present narrative review aimed to shed further light on the matter, accounting for both experimental animal models and clinical observations about major depressive disorder (MDD) pathology. *Method:* Major databases were inquired from inception until April 2016 for records about MDD and pain. *Results:* Pain and MDD are tightly associated with each other in a bi-directional fashion. Several cross-sectional and retrospective studies indicated a high presence of pain in the context of mood disorders, including MDD (up to 65%), but also increased prevalence rates in the case of mood disorders documented among people with a primary diagnosis of either psychological or somatic pain (prevalence rates exceeding 45%). The clinical implications of these observations suggest the need to account for mood and pain manifestations as a whole rather than distinct entities in order to deliver more effective interventions. *Limitations:* Narrative review, lack of systematic control groups (e.g., people with the primary diagnosis at review, but not the associated comorbidity as a study) to allow reliable comparisons. Prevalence rates and clinical features associated with pain varied across different studies as corresponding operational definitions did. *Conclusions:* Pain may have a detrimental effect on the course of mood disorders—the opposite holds. Promoting a timely recognition and management of such an often neglected comorbidity would therefore represent a primary goal toward the delivery of effective, multi-disciplinary care.

## 1. Introduction

The Diagnostic and Statistical Manual for Mental Disorders, Fifth Edition (DSM-5) [1] replaced the Fourth Edition (DSM-IV) [2] diagnostic criteria for “pain disorder” introducing new diagnostic criteria (“somatic symptom and related disorders”) sharing “somatic symptoms associated with significant distress and impairment” as main commonality [1].

Remarkably, this new disorder should be diagnosed “based on positive symptoms and signs (distressing somatic symptoms plus abnormal thoughts, feelings, and behaviors in response to these symptoms) rather than the absence of a medical explanation for somatic complaints” [1,2].

This later premise does not necessarily imply that pain disorders need to result in a DSM-5 mental diagnosis. However, whenever a somatic component (as opposed to an objective explanation) exists for chronic pain, then the “somatic component adds severity and complexity to depressive and anxiety disorders” eventually occurring altogether, resulting in higher overall severity, functional impairment, and even refractoriness to traditional treatments” [3].

Pain is likewise often associated with either Major Depressive Disorder (MDD) or Bipolar Disorder (BD), though not emphasized by the DSM-5 as the opposite eventual association [1].

This latter scenario, corroborated by the clinical practice, has significant implications, especially considering the high prevalence of mood disorders and chronic pain [4].

Depression is a leading cause of global burden and affects approximately 33% worldwide [5]. BD’s most conservative estimations indicate 2% prevalence rates among primary care patients [6]. People with depression and BD experience an inflated risk of multiple physical health comorbidities, such as cardiometabolic disease [7] and diabetes [8], which collectively account for the majority of the 15–20-year premature mortality gap documented in this population [9].

Overall physical healthcare is also poor among people with comorbid pain and mood disorders [10,11].

Nevertheless, pain is a significant ailment leading to medical attention by the patient [12]. The painful experience would represent a significant pathway to alert an individual about underlying pathology or distress against potential threats [13] and represent a complex evolutionary adaptation toward survival [14].

Pain and psychiatric disorder have been documented to be tightly associated with each other [4,15,16,17], with point prevalence rates up 50% of comorbid cases of MDD and pain [4]. For example, back pain and facial pain are associated with a 3–5-fold increase in prevalence rates of comorbid depression vs. pain-free controls [4,18]. Conversely, it has been observed that in patients suffering from severe burn injuries, anxiety, and depressive behavior are associated with increased pain rates at an ulterior moment [19]. Most distressing subjective pain and a high number of pain sites would correlate with a longer duration of untreated anxiety and depressive disorders as the suffering one would also ascribe affective symptoms to somatic pain [20]. Depression and anxiety are common among people with prominent pain [21,22,23]; BD would follow a similar trajectory [24,25,26]. When either type of pain associates with a mood disorder or the opposite association is neglected or left untreated for a long time, the potential consequences may be deleterious for overall well-being, including increased risk for suicide [6]. In contrast, the response toward antidepressant medications may be sub-optimal in poorly managed chronic pain [27,28].

The current narrative review aims to: (i) assess the prevalence and clinical features associated with the two-way association pain-MDD or pain-BD comorbidity and (ii) critically account for the collected evidence proposing guidance for increased diagnostic awareness and clinical management.

## 2. Materials and Methods

The present narrative review was conducted in accordance with standard procedures described elsewhere [29].

### 2.1. Eligibility Criteria

Studies documenting cases of (i) any mood disorder (including MDD, BD); (ii) measures pain in clinical and experimental conditions in human patients; (iii) measures of pain reactions in animal models of mood disorders were accounted for inclusion in the present review. There were included records of adult subjects aged 18 and over, of both sexes, diagnosed with BD, either BD-I, BD-II, or BD-Not otherwise specified (BD-NOS), or MDD. Diagnostic definitions were then to be based on either DSM or International Classification of Diseases ICD. No restrictions were inflicted in regard to the location of the individuals diagnosed with these affections (population or hospital-based subjects). Studies are not considered if they were either: case reports, conference proceedings, letters to the Editor, or reviews. Studies published in languages other than English were excluded.

### 2.2. Information Sources and Database Searches

The inquired databases (from inception till April 2016) were: ScienceDirect, PubMed/Medline, Scopus, PsycINFO, and Cochrane library. The keywords used in the search strategy were “pain”, “mood disorder”, “bipolar”, “depression”, “animal models” found either in the title or abstract or among the keywords section. Cross-references of included articles were also considered. The adopted PubMed string was: (((mood disorder[Title/Abstract] OR bipolar[Title/Abstract]) OR depression[Title/Abstract]) AND pain[Title/Abstract]) AND animal model[Title/Abstract].

### 2.3. Study Selection

Two independent authors (IA and AC) screened the titles and abstracts of the retrieved result of the search; only the ones potentially meeting the eligibility criteria were screened for a full-text review. For the full-text review, two authors established the final list of included studies. Any discrepancy was solved by consent with a third (MF) author.

### 2.4. Data Synthesis

Due to the paucity and heterogeneity of studies identified, a formal quantitative meta-analysis was not possible. Therefore, we employed the best evidence synthesis to identify the key results and disentangle the relationship between pain and mood disorders.

## 3. Results

### 3.1. Study Characteristics

There were 51 results generated by the PubMed search and 179 in Scopus. 31 additional papers were retrieved through searches in databases such as Cochrane, PsycInfo, or manual search.

### 3.2. Quality Assessment

There was an assessment of the quality, original studies being ranked according to Downs and Black criteria [30]. Details of the analysis of the quality could be found in Table 1 and Table 2.

### 3.3. Pain in BD

A cross-sectional epidemiological study made in Singapore on 6616 participants, aged 18 and more, with BD assessed by World Mental Health Composite International Diagnostic Interview version 3.0 (CIDI 3.0) indicated a BD lifetime prevalence of 1.2%, with a 40.4% (SE = 6.9; OR = 3.0) chronic pain comorbidity. Concerning the lifetime prevalence, BD-I hit 1.1% and 28.3% of chronic pain, BD-II 0.06% lifetime prevalence wit 59.3% chronic pain encounters, making chronic pain top prevalent comorbid condition [31]. Noteworthy is that in a retrospective study from the US including a total number of 227,691 participants out of which 4310 were diagnosed with bipolar disorder (1.9%) in- or outpatients, mean age 53 (SD = 13),10% female, compared to a VA group (*n* = 3,408,760), 10% female, mean age 58 also indicated that chronic pain such as lower back pain conditions was more prevalent in BD group (15.4%) compared to sample group (10.6%; *p* < 0.0001) [32].

### 3.4. Pain in MDD

In the view of current research, pain and depression are found simultaneously in 30 to 60 % of the cases [4,33]. Chronic pain is described as persistent or sustained pain that is encountered for a time frame of three months at least [34]. Despite attempts to consider chronic pain an individual disease, this can only happen if a new and particular pathophysiological pathway is described [35].

According to the existing research, depression is frequently correlated with chronic pain, presenting a rate of 65% of patients known with a depressive disorder who report pain manifestations [4]. Corruble and Guelfi, in the naturalistic observational study which implicated 150 inpatients diagnosed with unipolar major depression with the means of DSM-IV, ages varying from 18 to 70 (74% being females), reported at admission a prevalence of 16% patients with one pain complaint, 39% with 2 or 3 pain complaints and 37% with 4 or 5. Pain complaints were assessed through a Symptom Checklist comprised of 90 items—revised version (SCL-90R). With a frequency of headaches (70 vs. 51%, *p* < 0.05) and chest pain (55% vs. 38%, *p* = 0.01) higher in women, while men scored higher in myalgia(80% vs. 62%, *p* = 0.05) and numbness (57% vs. 48%, *p* < 0.05), baseline pain global scores indicated no relationship with depression severity registered through MADRS (r = 0.09); however, as the SCL-90R indicates they were connected to depression (r = 0.42, *p* < 0.0001) and anxiety (r = 0.50, *p* < 0.000) complaints [36].

A review paper following prevalence rates of pain in patients suffering from depression (diagnosed by a specialist) indicates at a rate varying from 21% (*N* = 161) to 76.5% (*N* = 51) in studies where the methodological means of pain manifestations are specified, but it ranges a lot more from 19% (*N* = 224; psychiatric inpatients) to 100% (*N* = 16; non-patients with multiple applied rating scales and psychiatric interview) if all the studies are considered, even if the methods of assessment are not specified [37].

### 3.5. MDD in Pain

There is a representative number of researches emphasizing the fact that people suffering from chronic pain develop depression and anxiety disorders at larger scales in comparison to those without the chronic painful complaint [24,38,39]. In 2008 a cross-sectional analysis by Bair [21] conducted through analyzing data from a previous study including 500 American patients from primary care (55% women; mean age 59 years) who all suffered from musculoskeletal pain reported that 250 participants met the criteria for clinical depression assessed through Symptom Checklist 20 (SCL-20), 76.3% of the clinically depressed ones having major depression. Regarding pain-related outcomes, for mainly pain severity and interference accounted for through Brief Pain Inventory (BPI)-Short form there was recorded that from the four clinical groups (pain only-p; pain and depression-PD; pain and anxiety-PA; pain, depression, and anxiety-PDA), the PDA group had the most significant pain severity and pain interference (F value= 5.88; df = 2.482; *p* = 0.003) [21].

In a group of 171 outpatients diagnosed with non-malignant work linked to pain experienced in the last 3 months—87 men (50.9%) and 84 women (49.1%), aged 19 and above (mean age 42.5 years), the depression mean rate registered with The Center of Epidemiologic Studies-Depression (CES-D) scale presented at 24.69 and on the Zung Self-Rating Depression Scale at 50.08, values above the normal ones of 16, respectively 50. Moreover, the prevalence rate of individuals who scored above the depression limit indicator was 60.2% in the case of CED-S marker and 64.3% in the case of the Zung scale. Furthermore, there was a reported prevalence of 28.1% of past depression states and even 35.1% of persons with a history of one or more of the following conditions: depression, anxiety, and substance abuse. Following the results registered with VAS (53.09; SD = 20.20) and MOS-PS (67.9%-daily or almost daily pain encounters in the last 4 weeks) scales, it was noted that 73% suffered from chronic pain according to the definition of the International Association for the Study of Pain Subcommittee on Taxonomy, while 26% were diagnosed with acute pain [40].

Other evidence indicates a correlation of depression (quantified through Diagnostic Interview Schedule-Version-III-Revised applied by a trained interviewer) with chronic pain manifestations, as presented in a study involving 71 females with fibromyalgia (non-patients and out-patients; diagnosed according to American College of Rheumatology criteria for primary fibromyalgia syndrome); among them, the ones who expressed higher current depressive symptoms (assessed through the seven-item Depression subscale of the Brief Symptom Inventory; mean = 0.59, SD = 0.57, *p* < 0.05) accuse greater daily pain (B = 14.77, F(1,66) = 9.08, *p* < 0.01) [41].

However, in another review report, it is recorded that depression rates vary across pain patients from 25% (*N* = 100) to 100% (*N* = 53) prevalence scores in studies where the methods of depression assessment are defined [37].

### 3.6. BD in Pain

Benefitting from information offered by applying the National Comorbidity Study-Replication Study (NCS-R) that implicated 9282 of participants located in the US aged 18 or older, it was reported that lifetime prevalence of chronic spinal pain, assessed by the use of NCS-R, was more frequently encountered in people diagnosed with a mood disorder (45.5%; SE = 2.2) by means of NCS-R, in contrast with humans from the general population (25.5%; SE = 1.0). Remotely, from the mood disorder category, bipolar I or II was ranked at a prevalence rate of 4.4% (SE = 0.6) in the past 12 months [24].

These aspects are summarized in Table 1.

### 3.7. Animal Models

Although there is no perfect replica of the human disease manifestation, animal models have been employed in an attempt to discover the mechanistic elements of illnesses and test possible methods of treatment in different maladies [46].

#### 3.7.1. Depression in Pain—An Animal Model

Zhou and his team studied an animal model of neuropathic pain, namely spared nerve injury (SNI) induced in mice and followed the rates of indoleamine 2,3-dioxydase-1 (IDO1) enzyme, which metabolizes tryptophan involved in the inflammatory process, cerebral inflammation being a demonstrated process with a role in depression [47] as compared to sham mice (*n* = 6–20 in each group). It was observed that SNI determined depressive-like behavior pointed out by the results of mechanical allodynia (registered through 50% withdrawal threshold employing von Frey filaments) in the hind paw where the surgery was practiced (surgery x time interaction: F(7,63) = 8.39, *p* < 0.01), and also time decrescence of social interactions with a conspecific adult (t(17) = 3.03, *p* < 0.01), along with a rise of immobility time in the forced swim test performed in the seventh post-intervention day (t(31) = 3.89, *p* < 0.001), but without the interference of SNI with spontaneous locomotor activity. Regarding IDO1 activity, no Ido1 mRNA expression was generated by SNI model in either the frontal cortex nor in the lumbar dorsal horn, but significant Ido1 mRNA activity was registered in the liver (IDO1:t(18) = 2.17, *p* < 0.05). Also, comparison with genetically modified mice with deleted Ido1 was performed (*n* = 8–15 per group) in day 5 after surgery, noting that the social interaction time in the SNI groups of mice without Ido1−/− and in the group with registered significantly decreased social exploration time (surgery × genotype, F(1,29) = 4.92, *p* < 0.05), although SNI did not interfere with social exploration in Ido1−/−mice. Increased immobility time was recorded between SNI mice without genetic alteration and with (surgery × genotype, F(1,49) = 8.25, *p* < 0.01); however, Ido1−/−mice did not present any raises in the period of time spent in immobility in the forced swim test 7 days or 3 weeks after SNI (surgery × genotype, F(1,10) = 6.06, *p* < 0.05) compared to the non-Ido1-/-mice. Noteworthy is that mechanical allodynia accounted at 7 days (surgery × time, F(4139) = 50.0, *p* < 0.01) or 3 weeks after SNI intervention did not modify in genetically tempered mice (surgery, F(1,21) = 1106.8, *p* < 0.01) [48].

In contrast to Zhou’s findings, Kim and his team demonstrated that IDO1 is also involved in pain along with depression in inflammatory conditions by demonstrating that inhibition of IDO1 activity through administering IDO1 inhibitor L-1-methyl-tryptophan (1-MT; 10 mg/d; intraperitoneally, twice daily for 14 days) in an induced model of an arthritic rat (hind paw monoarthritis- injection of 50 µL CFA) simultaneously reduces mechanical nociception (test: Von Frey filaments; F(3119) = 11.33; *p* < 0.05) and depressive manifestations (improved immobility time in forced swim test; F(3,63) = 5.54; *p* < 0.05) compared to sham rats (50 µL incomplete Freud‘s adjuvant) injected with vehicle control (Mean ±SEM, *n* = 6, *p* < 0.05) [49].

Another study noted that by inducing neuropathic pain through partial sciatic nerve ligation (PSNL) in male Swiss mice, there were observed changes in the parameters of the forced swim test, renowned for determining depressive manifestations. Data indicates that PSNL modified the latency time in forced swim test by decreasing it (21.5%), while the immobility time incremented (50%) compared to sham mice, indicators of depressive-like features. Evaluation of mechanical pain showed the presence of significantly higher frequency response to VFH stimulation in the ipsilateral paw where the surgical procedure of PSNL was performed compared to controls (t(12) = 30.67, *p* < 0.001). Consequently, it was noted that the contralateral paw of PSNL mice also presented increased sensitivity to VFH test, a phenomenon known as “mirror pain” (t(12) = 2.30, *p* < 0.05) [50].

Again, all these aspects are also summarized in Table 2.

#### 3.7.2. Pain in Depression Animal Models

Following reactions to neuropathic pain induced through spinal nerve ligation (SNL) in a rat model of depression induced through the well-validated procedure of olfactory bulbectomy (OB +SNL - *n* = 11), changes in nociception mechanical (von Frey test) and cold perception (acetone drop test) have been recorded via comparison with pre-SNL measurements. Before the induction of neuropathic pain, the rat model of depression proved to be sensitive to cold stimuli (decreased latency time in the acetone drop test: lick, shake, or withdrawal of hind paws; t34 = 2.15, *p* = 0.039), along with a slightly increased withdrawal frequency (U = 103.5, *p* = 0.06) compared to non-OB rats (*n* = 8–10), while also presenting with mechanical allodynia indicated by a sharp decrease in the 50% mechanical withdrawal threshold as opposed to sham-rats (t34 = 2.71, *p* = 0.011). Hargreaves test pointed out no significant response to heat nociception stimuli in OB rats [56].

When SNL was performed, mechanical and cold allodynia were registered in sham and OB rats in the ipsilateral hind paw, without heat hyperalgesia. OB rats subjected to SNL and tested for cold nociception parameters presented with a decreased latency time of paw withdrawal (t20 = 3.21, *p* = 0.005), along with a reduction of withdrawal frequency (t19 = 3.21, *p* = 0.005) in the ipsilateral hind paw compared with sham-SNL rats, proof of modified nociceptive response to cold stimuli. In this way, OB rats compared to non-OB presented with increased pro-inflammatory activity in the amygdala (↑IL-1β), along with raises in the mRNA expression of CD11b and GFAP (markers of microglial and astrocyte activation), although this increased inflammatory activity was not recorded after SNL procedure on OB rats. However, SNL induced a reduction of IL-6 (SNL: F1,23 = 21.4, *p* < 0.01) and increased IL-10 in non-OB rats with SNL (SNL: F1,24 = 10.5, *p* = 0.003) [56].

In an animal model of depression induced through unpredictable chronic mild stress (UCMS) it was noted that reaction to acute pain measured through paw withdrawal latency to an applied radiant heat to the left hind paw in the depression phase of UCMS was increased (UCMS rats 14.15± 0.36 s vs. controls 10.00 ± 0.49 s, *p* < 0.00), but in the recovery phase the latency period returned to normal (10.42 ± 0.43 s vs. control 10.45 ± 0.40 s, *p* = 0.96). Proceeding with a test for persistent pain through formalin administration, it was noted that behavior indicating pain was heightened in rats previously exposed to UCMS compared to controls as follows: in phase I 56.06 ± 2.79 s vs. 41.96 ± 4.20 s, *p* < 0.05, in interphase 22.7 ± 2.77 s vs. 13.98 ± 2.01 s, *p* < 0.05, in phase II, 407.6 ± 36.67 s vs.211.1 ± 35.91 s, *p* < 0.01) [57].

### 3.8. Evolutionary Hypothesis

Nowadays, current theories view depression as an evolutionary advantage. This theory is based on the fact that depression, despite its significant deficiencies regarding survival rates and reproduction, is seen as a mechanism of resistance against infectious processes [58]. This inflammatory background of the depressive disorder is supported by a significant number of researches, among the first ones being those performed by Maes and his collaborators [59] who also suspected the implication of cell-mediated immune responses [60,61] and demonstrated a connection between hyperactivation of HPA axis as a response to high inflammatory activity [62,63]. It was also demonstrated that serotonergic changes present in depression are due to an immune activation owed to cell-mediated processes [64,65].

Therefore, inflammation and cell-mediated immune activation play an essential role in developing depression, which in turn leads to the specific behaviors encountered in this affection. It is hypothesized that symptoms of depression act as an enhancer of the immune system through energy conservation and resource scheduling and limiting interaction with other people in this way, benefiting from the prevention of spreading infections [58].

Other advantages that depression brings according to the evolutionary theory are limited exposure to additional potential infectious agents, diminishes in the individual’s interaction with situations that might perturb the immune system from focusing on fighting existing infection, an indication of the potential contagious risk that the person suffering from depression might possess, in order that close ones will avoid contact [58]. Although the arguments in favor of depression being an evolutionary asset such as beneficial changes in reduced physical activity helping decrease the risk of infections and also incrementing the immune performance through a conserving energy mechanism, the demonstrated potency of antidepressives to combat infectious diseases are to some degree contradicted by Hagen who sees features as low mood and sadness as evolutionary traits, but characterizes MDD as dysfunctional sadness [66].

By the same evolutionary token, pain in the context of MDD is regarded through the psychic pain hypothesis where sadness and low mood play the role of evolved functions as a response to social adversity similar to how physical injuries provoke somatic pain answers [66].

Hagen emphasizes in its critical review on the thin line between depression as a pathological approach to adversity and sadness and low mood as physiological coping mechanisms, therefore highlighting the necessity of defining and understanding the sadness and low mood as normal reactions in comparison to the dysregulated mechanistic that provoke depression occurrences. Also, it is stated that the inconsistency appears when comparing subjects that suffer from depression to healthy individuals, rather than individuals equally exposed to affliction [66]. Therefore, this can be extrapolated to the situation when pain evaluations are made in individuals suffering from depression. There should be compared the reactivity to the pain of individuals diagnosed with depression versus healthy controls experiencing different adverse circumstances for the results of the evaluation to be accurate.

Pain and depression also share the biochemical background of activated inflammatory responses. The question is whether depression initiates the pain response as a result of inflammatory system activation, or is depression induced through pain occurrences?

By analyzing the biochemical processes activated by depression, it can be noted that amongst them, there are pro-inflammatory responses and activation of immune cells; one might assume there is a connection between all these mechanisms and pain occurrences.

The presence of inflammation in the context of depression is indicated by multiple signs such as increased levels of IL-1, IL-6, TNF-alfa [67]. Pain occurrences in the form of chronic pain are also characterized by incremented pro-inflammatory cytokine levels positively correlated with pain intensity and decreased levels of anti-inflammatory cytokines (such as IL-10) inversely correlated with pain intensity [68,69]. On the other hand, nociceptor neurons express receptors for these pro-inflammatory cytokines (IL-1, IL-6, TNF-alfa), leading to their activation. This, in turn, generates activation of second messengers Ca2+ or CAMP, causing an activation signal to several kinases (PKA, PKC, PI3K, MAPKs). This event causes hypersensitivity and hyperexcitability of nociceptor neurons, thus leading to increased pain signals coming from the periphery [70]. Therefore, there might be a possible reason behind the association of pain with depression.

As observed from the peripheral blood, psychic stress leads to the activation of inflammatory pathways conducted to increased interleukin-6 (IL-6) and other pro-inflammatory cytokines. Of note, all these manifestations indicate that the organism initiates an immune reaction to counteract a threat to the individual’s psychiatric features (emotions) and, surprisingly, not to an external threat such as a pathogen. The reason is theorized to be an evolutionary mechanism through which humans, on account of traditional immunological checks and balances that were previously made in modern times, lack previous inflammatory threats that would activate ancestral immunological and behavioral features accountable for high rates of various inflammatory disorders [71].

There are also genetic theories supporting a genetic alteration (for instance, 5-HTLPR short allele) as a cause for distinctive exposure to negative emotions of the subject expressing this specific genetic pattern [72]. Moreover, exacerbation of pain experience has been correlated to the incapacity of the individual to overcome negative feelings [73]. In these situations, pain catastrophizing (overmagnifying pain experience) is often reported to increase the risk of depression [74].

Another point of view regarding the origin of depressive dysfunction incriminates glial abnormalities. Formerly, it was demonstrated that part of the effect of antidepressants is due to their stimulation of astrocytes activity and density [75]. Decreased connexin 30 (Cx30) immunoreactivity in the prefrontal cortex was correlated to the presence of MD in a post-mortem study in humans [76]. In the same way, it was demonstrated that exposure of rodent animals to unpredicted chronic mild stress leads to essential changes in expression and function of Cx43 gap junction channel, both cortical and hippocampal. Furthermore, Cx43 astroglial levels are increased by the administration of typical antidepressants (fluoxetine, duloxetine) or tricyclic antidepressants (amitriptyline) [77,78].

Moreover, Cx43gene was proved to be involved in mediating trigeminal pain. It was demonstrated that by reducing Cx43 expression, pain-like behavior was reduced while overexpression of Cx43 induced pain-like behavior [79]. Therefore, there might be another explanation for the frequent complaints of pain encountered in depressive manifestations.

## 4. Discussion

### 4.1. Depression and Pain vs. Pain and Depression

#### 4.1.1. Hypothesis

Literature’s findings on the connection between pain and depression appear to contain three main hypotheses that explain this link [4,80]. One hypothesis implies that pain is a result of depression [81]. This idea is sustained by an international study where 69% of people suffering from major depressive disorder (MDD) presented with somatic symptoms, most of them being pain-related [82]. Moreover, in another research that included several 150 patients, it was noted that 92% signaled the presence of minimum one pain condition, and a rate of 76% encountered several pain sensations [36].

Another hypothesis advocates for the idea that depression is provoked by pain [83,84,85]. Moreover, a third theory indicates that depression and pain have a bidirectional connection [86,87,88].

Beyond being considered a predictor factor of pain or the other way around, there is another opinion that argues for the equivalence of pain and depression, affirming that chronic pain is in fact, a type of depression [89]. Evidence indicates that pain and depression share similar brain zones implicated in mood regulation and affective pain constituents [90]. From a biological point of view, it appears that these two conditions have common pathways and neurotransmitters [4,91].

A new theory that tries to explain the pathophysiology of depression occurrence bases its approach on mitochondrial dysfunctions. For instance, in a study where it examined the process of respiration at a mitochondrial level through high-resolution respirometry, there were registered significant differences between patients with major depressive disorder and without. The study was conducted in a group of 22 females diagnosed with major depression as compared to 22 females recruited as controls. From the group with MDD there were displayed decreased basal and maximal respiratory levels in the mononuclear cells found in the peripheral blood in comparison with the healthy ones [92].

Although these are promising results, it must not be omitted that a majority of participating patients were enduring antidepressant or antipsychotic therapy [92]. Therefore, the hypothesis must be tested in naive to treat persons diagnosed with MDD.

A significant correlation between depression and a specific type of pain, namely affective pain quantified through a short version of the McGill Pain Questionnaire, is revealed by a Canadian study involving 185 patients suffering from different injuries manifesting chronic pain where the prediction of returning to work potential is recorded. Following several specific profiles described in the study, it was observed that moderate depression, along with high affective pain, was considered as the culprit for the deterioration of returning to work percentage (18–21%). Therefore, the combination of pain occurrences and depression does negatively influence health outcomes and drove the authors to sustain the hypothesis of common pathogenetic mechanisms where serotonin and norepinephrine neurotransmitters are blamed for direct and indirect involvement in both affections [45].

Another incriminated factor in the etiopathogenic of depression and other mood disorders is oxidative stress [93,94,95,96,97], a process that appears when the balance between the physiological process of oxidation and reduction reactions is impaired. According to Can and collaborators, in research including 71 mood disorder patients and 30 healthy peers, it was observed that serum superoxide dismutase and glutathione peroxidase levels, two antioxidant defense enzymes, were decreased, indicating in this way, the presence of incremented rates of oxidative stress. Also, malondialdehyde rates were significantly increased, suggesting high lipid peroxidation activity, resulting in decreased antioxidant defense levels in mood disorders [96].

Interestingly, oxidative stress is also implicated in pain processes as it was previously demonstrated by our group, which showed a significant correlation between oxidative stress and the nociceptive process of the renin-angiotensin systems a result of angiotensin II and angiotensin-(1–7) administration [98,99]. Furthermore, considering that papers are advocating for changes in the oxidative stress status in persons with different types of pain and also knowing that mood disorders frequently coexist with pain [100], oxidative stress is an element incriminated in both conditions: pain and affective disturbances. For this reason, it might be a predictive factor of the duet pain versus mood disorder, but with unexplored and, therefore, new traits and features.

#### 4.1.2. Clinical Implications

The joint presence of pain and depression, approximated by 30% to 50% [4,101,102], leads to increased morbidity and also increased occurrence of poor treatment if one of the affections is omitted [103]. Following the research outcomes of Kroenke and colleagues brings into attention the necessity of evaluating pain and depression as an ensemble rather than as separate conditions in the clinical management of these illnesses and also in experimental conditions. Recognition of their presence and severity features should be evaluated as an interdependent relationship, as opposed to individual rating. Moreover, the existence of one condition should require an examination of the other [103]. This statement is backed up by reports that indicate a higher disability rate when both pain and depression are together in comparison with the occurrence of a singular affection [4,104]. Also, it was observed that pain encounters might decrease the outcomes of therapies against depression and, as well, depression might interfere with pain treatments by reducing their effectiveness [4,101,105].

Adding up the prior findings, conclusions of Kroenke’s study highlight the bidirectional relationship of the pain-depression dyad and a probability of a causative link between each other, accentuating on the importance of diagnosing both conditions, otherwise there is a high possibility of low-quality treatment response occurrence [103]. Contrary to the majority of studies incriminating pain as a factor for the occurrence of depressive-features, von Korff and Simon in their review paper, indicated that chronic pain does not have a specific relationship with depression, but rather is correlated with a variety of psychological conditions. This observation was made based on the Who Collaborative Study of Psychological Disorders in Primary Care during which persistent pain was evaluated through Composite International Diagnostic Interview-Primary Care Version, in a number of 5381 patients, and it was most encountered in individuals suffering from both depressive and anxiety states (47.2%) as opposed to those with no disorders (8.6%) and with no significant differences between individuals with either anxiety or depression. Even more, in the same review, it is emphasized on a population-based prospective study where chronic pain such as back pain, headache, and temporo-mandibular joint pain is followed, noting he lack of significant association between pain intensity and depression, but as the pain became more diffuse, significant increases in depressive behavior were recorded [86].

Another noteworthy aspect is that the relationship between pain and depression is shaped by the time factor, observed among 266 patients in primary care with back pain manifestations that depression did not increase levels in the situation of patients with unimproved back pain symptoms, only to see that after 7 weeks, patients with unimproved back pain presented with significantly increased depression levels, leading the authors of the review paper who analyzed the study to affirm that the transformation of pain in a chronic state was not the reason of increased depression but rather the event of an incomplete recovery. Also, a follow-up study indicates that depression rates did not have an ascendant tendency passing the time, but rather maintained their trend with even if the pain dysfunction continued, as it was a review in von Korff and Simon’s paper [86].

A critical perspective over the studies following pain and depression instated in a review paper argues that methodological procedures have significant imperfections, which leads to various results. The issues highlighted are varying limited form use of recognized diagnostic criteria to lack of specifying the methodology of pain or depression assessments, no differentiation between the types of pain assessed, missing control group for comparison. Despite all these discordances found and highlighted by Romano and Turner, a notable aspect that they pointed out is that in 50% of the individuals’ pain and depression act as a simultaneously occurring event. Also, it was noted that 40% of the patients accused depression just after pain occurrence altogether. If these remarks are confirmed, proof of the existence of a minimum of two separate subgroups of these individuals is uncovered [37].

According to a study conducted by Ang and his team where they followed the connection between fibromyalgia pain and depression, they observed that the presence of depression had a negative influence on pain as well. By using nociceptive flexion reflex (NFR), an assessment tool of spinal nociception utilizing electromyography as a measurement of the electric stimuli applied to the sural nerve of the biceps femoris muscle; they demonstrate that present pain and depression were autonomously affiliated with lower NFR threshold. This means that amplified nociceptive responsivity was recorded [106].

When the reaction of patients with fibromyalgia pain and comorbid depression was tested, it was noticed that the psychiatric condition appears to decrease the association of clinical pain and NFR threshold. An association of the two factors—clinical pain and NFR threshold was found in the non-depressed patients’ group but not in the depressed ones [106]. In this way, it looks like depression alleviates the connection of current pain and NFR thresholds, indicating the possibility of a central sensitization mechanism involvement [107], defined by the International Association for the Study of Pain as an increment of responsiveness in the central nervous system, specifically of nociceptive neurons.

In opposition to the general opinion, an interesting pain study conducted in patients suffering from depression proved that the thresholds for experimental pain were mainly augmented in a majority of stimulation situations, signifying that people experiencing depressive symptoms are less sensitive to pain induced stimuli [108].

Nonetheless, other researchers indicate differently. In one of the studies, it was described that pain thresholds and also pain resistance was decreased in the group of persons diagnosed with depression before treatment was instituted [109]. Moreover, in another study, it was declared that for a specific type of pain (lower back pain), depression was considered a risk factor with a ratio of 2:1 [110].

In regard to the origin of pain and mood disorder connection, a joint neuroanatomic substrate is implicated in mood processing and pain interpreting. It appears that they share specific brain regions, and therefore transformations occurring in these areas provoked by chronic pain might result in modifications of affective information, creating the conditions for mental disorder occurrence. The anterior cingulate cortex (ACC) and the insular cortex are assumed to be incriminated in both processes of pain and mood disorders [111,112,113].

A recent study demonstrated the crucial role that ACC plays in pain processing and storing antidepressive reactions to chronic pain. Furthermore, the study highlights the significance of the ACC role in mood disorders. On the contrary, the role of the posterior insular cortex was not demonstrated, appearing to have an influence only in mechanical allodynia [114].

Meerwijk and collaborators also explored the link between depression and psychological pain. This type of pain is known to be a frequent feature of clinical depression [115]. This type of pain is known to be a frequent feature of clinical depression [116], and it originates from the inability of patients with depression to satisfy primary requirements, which can lead to sensations of impaired abilities regarding work, family, and social experiences. Their research focused on exploring the frontal resting-state brain activity frequently involved in depression by employing electroencephalography (EEG) measurements. So, their findings indicate to low-frequency transcranial stimulation as constituting a possible solution to amend psychological pain [115].

Catastrophizing is highlighted as another mechanism that might explain the correlation between pain and depression, which was found to play a central role in both depression and pain, leading to the assumption that it might be the junction element between the two of them, as the authors of a review paper observed. Another one is emotion regulation, considering that each condition can be an equally powerful emotional stressor. The authors of the review conclude that depression and pain have a bidirectional relationship, affecting one another, and are also involved in the persistence and evolution of chronic conditions [117].

Although depression and pain share a number of common mechanisms, there are still differences recorded between them. It was observed that patients with depression differentiate themselves between those searching treatment in the psychiatric field and those looking for pain alleviation. Therefore, Linton and Bergbom pointed in their review paper, to different studies accentuating on these symptom differences between patients suffering from depression in a psychiatric context, who often present with feelings of hopelessness, worthlessness, accompanied by suicidal thoughts, while, on the contrary, patients suffering from pain phenomenon rarely are characterized by these traits, being mostly concentrated on feeling blue and the repercussions that pain brings [118]. Another research sustains the idea that the differentiation between the two groups resides in the blame side the patients choose. While psychiatrically assessed patients appoint blame to themselves, patients with pain that might experience depressive symptoms blame others [119].

Having in mind that despite the decades of studies dedicated to the pathophysiology of MDD, depression treatment methods are effective in only 30–60% of the cases [120] and adding-up the influences that the pain process appears to possess on mental health issues, the possibility, and necessity of developing new therapeutic approaches is appealing and impairing.

### 4.2. Bipolar and Pain vs. Pain and Bipolar

In the current literature, a special connection has been recorded between bipolar symptomatology and pain manifestations, leading to a captivating framework between the two of them.

Lately, another study conducted in Singapore, covering the prevalence of chronic pain in the context of bipolar disorder, indicated that 40% of patients with this psychiatric condition declared chronic pain manifestations [31]. Also, in a group assigned to an American government community for the elderly, from where it was considered only a sample of bipolar suffering patients, it was recorded that lower back pain is more frequently detected than in other individuals [32].

Moreover, according to Birghenheir’s study, bipolar diagnosed persons were correlated with higher exposure to painful experiences, excluding cancer pain in a group of older adults belonging to the Veteran Health Administration System compared to veterans without the psychiatric illnesses [121]. These results fit prior findings highlighting the high probability of a simultaneous pain condition in a person diagnosed with depression [4,38,39].

The explanation behind the strong connection between pain and bipolar disorder might be due mostly to the occurrence of depressive features in persons with the bipolar condition [121]. Birgenheir‘s study also states that the co-occurrence of bipolar and painful experiences is not because of either covariate entirely but seems to be autonomously linked to the high potential of chronic pain circumstances amongst veterans [121].

A unique view over the matter of bipolar illness and its connection to chronic musculoskeletal pains is brought by Wallace et al., [122], who are hypothesizing that these overlapped symptoms might actually be a form of pseudo-fibromyalgia. Fibromyalgia frequently termed ‘pain without purpose’ is co-morbid with BD in 95% cases [123].

Noteworthy is that the patient‘s psychiatric condition before an intervention that might cause physical distress, also pain, is influent in regard to the severity of pain felt by the patient [124].

Adding-up, there is also epidemiological analysis that describes subjects suffering from BD are at a higher possible risk of suffering from pain interference compared to those without. Even more, this study reported a higher prevalence of pain interference among patients with BD as opposed to those with anxiety or MDD [125].

In a longitudinal analysis of data following patient’s perception of physical health in the context of BD, it was concluded that most perception of deficient somatic health was afflicted with worsening of illness symptoms along the 24 months the patients were taken under observation for the study [126].

Therefore, because of the interferences of one’s thoughts or attitudes on the course of the illness and its treatment, it is important to include these affective and cognitive sides also in the therapy diagram.

### 4.3. Animal Models

The hypothesis of inducing mood disorder via chronic pain is supported by experimental data using chronic pain animal models that have the capacity to induce anxiety-like traits and/or depression-like features in the studied animals depending on the time variable [110,127,128]. Also, clinical data incriminate chronic pain in inducing mood disorder, considering that patients with chronic pain have a mean rate of 50% exposure to MDD [129]; therefore, chronic pain is accounted as a risk factor.

Studying mood disorder and pain interactions proves to be a complicated task having involved a considerable amount of interfering factors, but by employing animal models developed especially for these conditions, many of these factors can be controlled. As described in a review study following neuropsychiatric animal models [46], there are several types of modeling methods correlated with the cellular dysfunctions causing the malfunction.

These methods play an important role in the development of understanding the connections between pain and mood disorder because the experimental environment can be attentively monitored and variables linked to pain and mood disorders specifically quantified.

Knowing that inflammation is almost always accompanying pain [130], inflammatory compounds and reactions should be considered to be monitored.

In this way, Zhou and his team’s work demonstrated on a mice population that peripheral indoleamine 2,3-dioxygenase 1 (IDO1) is implicated in depression-like behavior expression, but not involved in neuropathic pain [48]. This rate-limiting enzyme is part of an inflammatory process thought to be taking part in depression development as this IDO1 mediates activation of pro-inflammatory cytokines and participated in the production of kynurenine and its metabolites in the brain hippocampus area [131]. Although clinical circumstances incriminate tryptophan/kynurenine metabolism in pain appearance as observed in the correlation between higher plasma kynurenine/tryptophan proportion with increased pain degrees [132], the data pooled from Zhou’s experiment on animal models showed that IDO1 does not contribute to pain in low-level inflammation circumstances. On the contrary, the proceedings of Kim’s research demonstrated that by controlling hippocampal IDO1 through interleukine-6 it could be regulated depression-like behavior and also pain manifestations in rat models induced through persistent hind paw inflammatory pain [49].

A possible explanation for this difference in results recorded between Kim and Zhou experiments is given by the latter highlighting the different amounts of inflammation generated by the employed pain induction methods in the animal models, one using spared nerve injury [48] and the other, persistent hind paw inflammatory pain [49], which reside in different degrees of inflammation, the spared nerve model recording much milder inflammation [48]. Therefore, the role of IDO1 in pain context is still in need of documentation.

It was discovered that abnormalities in orexin/hypocretin neuropeptide system action in the lateral hypothalamus result in sleep disturbances, depression, and affect motor activities. Also, sleep perturbations have been recorded in the presence of pain phenomena, being implied even a reciprocal relationship between sleep and pain in the way that pain interferes with sleep, and the interference is causing even more pain [133].

Another experiment where inflammation is incriminated in the occurrence of depression and pain involves an OB model of depression with an SNL model of neuropathic pain where there were highlighted the presence of altered neuroinflammatory gene expression in the amygdala, a part of the brain known to be involved in both depression and pain events [56].

Considering the results of the partial sciatic nerve ligation model pursued by Gai, it can be observed that a clear depression-like behavior is registered, accompanied by sensitivity in perceiving mechanical pain, which shows once more the flagrant connection between chronic pain and depression. A noteworthy aspect is that the effect o F-DPS, documented to possess anti-depressive propriety in both acute and subchronic doses, only has an antinociceptive effect in a daily subchronic dose in both ipsi- and contralateral paws of the tested animals [50].

Although there are experiments,—such as the one of Hu and his team—that indicate comorbid depression in chronic pain as being a result of chronic pain syndrome and that antinociceptive treatment should be able to amend these symptoms, there are still clinical situations where depression is not present in chronic pain patients, which lead Hu to assume that other mechanisms are implicated [53]. Measuring the effects of two antidepressants on a model of neuropathic pain appears to have different influences on mechanical pain, duloxetine having an analgesic effect, a drop of mechanical hypersensitivity being recorded, while fluoxetine did not produce any effect on mechanical pain [53]. Considering that these two drugs belong to different anti-depressive classes, the lack of fluoxetine effect, a selective serotonin reuptake inhibitor, was explained by the combined action on both 5-HT1A and 5-HT3 receptors with simultaneously anti- and pronociceptive proprieties which might cancel out one another. On the other hand, duloxetine, selective serotonin, and noradrenaline reuptake inhibitor auctioning only on 5-HT and NA systems, leads to mechanical pain alleviation due to the co-adjuvant reaction [53].

Noteworthy is that in another experiment also involving a neuropathic animal model, paroxetine, also a selective serotonin reuptake inhibitor, registered a decline in mechanical pain perception in an acute dose and sub-chronic administration after 2 weeks [50]. This difference in reactions between antidepressants of the same class might be due to the distinct animal models employed and particular methods of drug administration.

Another significant factor implicated in the augmentation of pain in depression is the neuropharmacological mechanisms involved in modulating these pain behaviors and depressive-like former experiences. By inducing a model of depression through unpredictable chronic mild stress, it was demonstrated that although the response to acute pain was similar to the controls after the depressive-like behaviors wore out, the increased reaction to persistent pain did not modify even after the period of depressive-like effects past. Therefore, there were studied the effects of muscimol, a GABAA receptor agonist, once it was administered directly to the basal lateral amygdala (BLA) and to the medial prefrontal cortex (mPFC). Contrary effects were registered to the two administrations, the one to the BLA presented with reduced persistent pain and decreased depressive features in the UCMS rats while the administration to the mPFC showed increased nociceptive responses to the persistent pain and increased depressive features. This increment in persistent pain responses in rats with a history of depressive-like traits suggests the opposing roles that BLA and mPFC may express in the modulation of pain reactions after an elongated period of pre-exposed to stress rats [57].

Pursuing the mitochondrial dysfunction hypothesis, it was noted in animal models of depression that depressive-like behaviors registered in these models are associated with mitochondrial damage in areas of the brain such as the frontal cortex and hippocampus, known to be involved in the human depression disease [134]. For instance, in Rezin and collaborators studies where they used the chronic mild stress rodent model, it was observed that areas such as the cerebral cortex and cerebellum were implicated in the mitochondrial processes, but without the hippocampus, prefrontal cortex, or striatum [135].

It was hypothesized that there exists a difference in experiencing depression between genders but analyzed from a mitochondrial bio-energetics perspective; the current evidence does not support a gender difference [120]. This affirmation is supported by studies following rates of cellular respiration registered at the level of human platelets in both males and females with no recording of significant differences [136,137].

To summarize, it can be observed that animal modeling is an important tool in studying mood disorder deficits/mechanistic and their relation to pain perception.

### 4.4. The Relevance of Anxiety, Depression and Pain Co-Occurrence

It has been previously demonstrated that anxiety and depression co-occur in patients diagnosed with chronic pain [138].

The motivation behind pain, anxiety, and depression co-occurrence resides in the fact that pain and mood disorders such as anxiety and depression share neuroanatomical pathways and also dysregulation of the same neurotransmitters implicated in both pain manifestation and changes occurring in these disorders. In light of relatively recent research, it looks like the presence of both anxiety and depression conditions overlapped with pain influences pain outcomes. Bair et al. demonstrated that the assembly of all three conditions leads to more elevated pain feelings compared to individuals that suffer either only from chronic pain and depression or chronic pain and anxiety [21].

Among the influences cited in the occurrence of orofacial pain in the context of the temporomandibular disorder are psychological and psychosocial elements [139,140,141], alongside depression [142] and especially elevated anxiety rates [143].

There is a great amount of literature describing the correlation between anxiety and/or depression and functional abdominal pain (a medically unexplainable condition) recurrent in children and adolescents at a rate of 7–25% [144]. So, in the pediatric environment, it has been hypothesized that children are prone to somatization which might mediate the link between anxiety and depression with abdominal pain [145]. Also, emphasizing on the connection between pain versus depression and anxiety, von Korff reiterated in his review the outcomes of a WHO project accentuating the fact that co-occurrence of anxiety and depression increases previous persistent somatoform pain manifestation compared to individuals without disorders who are manifesting less of these somatoform symptoms, but anxiety alone and depression alone do not have the same significantly increased outcome of persistent somatoform pain reactions as co-occurring depression and anxiety [86].

As demonstrated by Lavigne and his collaborators, in a school-age community sample of children, there might exist a mediation relationship between the tendency to somatize and anxiety, depression, and abdominal pain [146]. According to Cho and his team, pain severity rates are correlated with the severity of depression and anxiety among people suffering also from chronic shoulder pain [147].

Also, in a group of patients scheduled for rotator cuff repair, a study conducted by Cho and collaborators admitted that depression and anxiety provoked adverse outcomes in concern to preoperative pain. As a consequence, it was highlighted the importance of evaluating pain intensity, depression, and anxiety levels of every person with attentive consideration before proceeding to the surgical intervention [148].

Altogether, the conjugation of anxiety, depression, and pain appears to have different dynamics in concern to the outcome reactions, as compared to only one affective trait and pain as well in comparison to non-psychiatric persons experiencing painful events.

### 4.5. Pain Predictability

An interesting observation is that anticipatory anxiety before medical procedures incriminated to evoke pain in children actually heightens the pain reaction [149,150,151]. Because children with recurrent abdominal pain expressed the same anxiety symptoms and somatic sufferance as the ones diagnosed with anxiety disorders [152], it was implied that anxiety and abdominal pain might in fact be a single disorder [153].

It appears that strategies adopted to cope with pain have an important role in determining the effects of this process. In this way, applying passive coping strategies as for example, self-isolation, catastrophizing, and activity avoidance resulted in augmentation of pain sensations, physical symptoms, depression, and anxiety rates [154,155,156,157].

Other intriguing findings uncover that persons with BD that are responsive to their therapy claim decreased levels of pain in comparison with non-compliant to treatment peers [158]. Also, the pain complaint in individuals diagnosed with BD was raised to four types of painful events at a time [159].

Another aspect is that although this group of individuals appears to be more exposed to the chronic pain experience, they are less likely to search for medical assistance [121]. Stubbs and his colleagues conducted a meta-analysis on pain prevalence in persons suffering from BD and uncovered that a large part of patients with BD complained of clinically significant degrees of pain [26]. The specific types of pain that prevailed were chronic pain, with almost one in four patients being affected, and followed by migraine, where one in 7 people had BD [26]. Findings of a recent meta-analysis demonstrated that fibromyalgia and BD are comorbidities, the prevalence of BD in the context of fibromyalgia being 21% [160].

Focusing on psychological state and pain perception experience, one study followed their evolution prior to and after surgical intervention in women with breast cancer. It was found that 27.1% of women declared breast pain before the surgical procedure. The group of patients who had not declared pain before, but experienced acute pain after the intervention, marked higher on anxiety and depression scales [161]. This leads us to believe that the absence of pain before the intervention might be a predictor of future possible affective perturbations.

Another study focused on the associations between pain and psychological status before women with breast cancer were scheduled for surgical procedures. Findings uncovered that 37.5% of women scored significantly high on the depressive symptoms evaluation scale, regardless of the pain. The same situation is repeated with anxiety levels, leading to the affirmation that a significant number of women scheduled to undergo surgical intervention for breast cancer are suffering from important psychological distress before the procedure [162]. Also, Kyranou and collaborators caution that women with past depressive experiences, scoring high on depressive traits and reporting pain before breast cancer surgery might be a negative predictor of postoperative results, considering that 31% of women who declared having pain manifestations prior to the intervention had a history of depressive symptoms when compared to the no pain group [162]. Therefore, pain experiences connect in one way or another to mood disorder manifestations.

An unexpected factor that contributes to pain and depression development is loneliness, defined as a social isolation state perceived by the individual experiencing it as painful [163]. People confronting this particular emotional state scored less abilities in tolerating somatic pain in comparison to those who were perceived to be socially integrated [164].

Altogether, it is noticeable that there are several unforeseen factors that can be incriminated as predictors of future pain experiences in the context of mood disorders. This observation might be useful in developing new strategies of fighting devious pain reactions encountered in mood disorder.

### 4.6. Neuroticism and Its Associations with the Risk of Affective Disorders and Pain Vulnerability

Lately, there is also an increasing trend in the current literature regarding the etiology of neuroticism, and also its connections with the risk of affective disorders and pain vulnerability, evidencing the mediator role of personality for pain and affective disorders [165]. Thus, neuroticism was described as a stable, genetically-based dimension of temperament, which can be viewed as a consequence or response to elevated stress, being related to the psychological profile of every individual and based on a multitude of endo- and exogenous factors fulfilling the role of amplifiers [166].

In this way, it was recently demonstrated that genetics can be fundamental in the connections that might exist between pain, depressive, and neuroticism’ phenotypes. Therefore, genetic correlations were performed for example, by linkage disequilibrium score regression method in a UK cohort that consisted of 151,922–226,683 individuals. By estimating the genetic correlation of 8 pain phenotypes with other psychiatric traits, Meng et al. [166] demonstrated that all pain phenotypes are heritable (h2 = 0.31) and they have a strong and positive correlation with other pervasive low moods by sharing to some extent a similar mechaniscistic pattern.

It was also hypothesized in two previous studies that pain as an overall construct is a state-driven by anxiety, but not by depression in adolescents [167,168]. Thus, anxiety partially mediates the relationship between anticipatory anxiety and this Big Five trait, without any interactions which are otherwise stable across fluctuating levels of depression. Thus, given the lack of evidence in which such an approach is assessed, neuroticism could be categorized as a vulnerability factor for elevated pain responses in both adolescents and chronic pain patients [167,168].

In order to offer a conclusive perspective of the aforementioned aspects, neuroticism can be considered a consequence of the pain and the related fear of both current and previous experiences, which are felt or perceived [169]. Concomitantly, it should be taken into consideration that pain catastrophizing is very much related to each one’s thoughts and type of thinking, being distinct from other measures of negative personality and affect [169].

Another particular feature was also recently described, with a description of a persistence tendency of memory for painful events up to 7 days [170], without significant differences in terms of sensory mechanisms alteration and depending on personality traits of neither extraversion nor neuroticism [171]. Also, neuroticism could act as a risk factor by predicting the course and severity of psychiatric comorbidities, such as major depressive disorder [172].

Recent evidence also showed that most genetic regions (25/37) associated with depression share a similar pattern with neuroticism [173]. In this way, depression was positively correlated with cardiovascular, metabolic, and psychiatric phenotypes and negatively with personality trait conscientiousness [173].

A similar pattern was observed in the case of the genetic associations between MDD and BD, polygenic neuroticism being significantly associated with MDD, while polygenic extraversion with BD [174]. Some similar discrepancies in personality traits were also observed, since, for example, Bipolar I Disorder patients tend to be less neurotic, as compared with individuals diagnosed with Bipolar II Disorder [174,175].

### 4.7. Possible Therapeutic Avenues

It is our belief that by understanding this intricate connection between pain phenomenon and mood disorder and raising awareness in regard to this viable existing link between the two of them, the psychiatric specialist will not overlook the somatic aspects of the mental disease, increasing the chances of reducing dimensions of the psychiatric features and vice versa.

As pointed out by a recent systematic review, the information in regard to oxytocin’s effect on pain manifestations in human and animal individuals is leaning on the potential use of this nano peptide hormone as an analgesic [176]. However, although the lowering of noxious stimuli was reported, the accuracy and stability of these results could not be evaluated [176].

Moreover, physical exercise has been pinpointed as a beneficial and protective method to reduce corticosterone production, a stress corticosteroid hormone involved in the appearance of depressive symptoms in rodent models [177]. Anxiety and corticosterone levels also have a strong relationship; repeated administrations of this stress corticosteroid in animal models were observed to create important emotional changes that stimulate anxiety disorders [178,179]. Also, knowing that pain increases plasma corticosterone levels [180], physical exercise might prove to be an efficient method to treat pain and mood disorder symptoms.

Sleep perturbations and lack of physical exercise expose individuals to pain problems, depression, fatigue, and decreased health status [181,182,183,184]. In this way, sleep issues also encountered in some anxiety disorders [185] and BD [186] might be an indicator of risk exposure to pain deficiencies.

Another possible aim that therapy should consider is loneliness. This is endorsed by the results of a study conducted by Jaremka in a population of breast cancer survivors where he pinpointed loneliness as a significant factor in predicting higher rates of pain, depression, and fatigue, as opposed to their less lonely peers [163]. The reason behind this association is awarded to immune dysregulation, incriminated to be the potential mechanism linking this triad cluster of pain, depression, and fatigue to the emotional state of loneliness. Along these lines, it was observed that lonelier individuals score higher cytomegalovirus antibody titers, knowing that this elevation signifies suboptimal cellular immune control over the dormant virus [163]. As mentioned before, another instrument that might prove to be valuable in the treatment of pain in depression mood disorders might be low-frequency transcranial stimulation, as pointed out by EEG explorations [115].

Considering all of the above, we echo with the view of Goldstein and his team, who sustains that pain should be methodically recorded during psychiatric interventions [125] and support the idea of undertaking pain management as a part of the psychiatric assessment process and monitor it over the treatment period. The benefits of this action serve on many levels, from the improvement of the pharmacological methods employed in mental treatment to raising the life quality of the patients.

Continuing with this concept, it should be also mentioned that BD and MDD also proved to be positively correlated with the overall functionality of the digestive tract [187]. This was due to the fact that ROME IV has offered a more conclusive perspective. More precisely, with the inclusion of affective and neurological components, any idiopathic cause indicates a functional gastrointestinal disorder (FGID) [188].

Thus, FGIDs such as irritable bowel syndrome (IBS), which is the most common functional gastrointestinal disorder, is recognized as a disturbance of the gut-brain (GBA) and hypothalamic–pituitary–adrenal (HPA) axis respect [188]. Despite the fact that IBS’ underlying mechanism of action is still obscure, it possesses a clear clinical panel, abdominal pain and/or discomfort and altered bowel function being predominant reported in patients [189].

In the literature relatively few studies can be found aiming to elucidate the interplay BD and/or MDD in the context of IBS [187]. However, as has been presented throughout the present manuscript, pain is elusive but also integrative criterium, which defines FGDIs.

It has been established on three distinct occasions that in patients suffering from MDD (onset or recurrent episode) the prevalence has ranged between 27–29%, up to 47,3% [190,191,192]. A cross-sectional study was conducted by Karling et al. [193]. On the other hand, those in remission did not differ from the control group in terms of gastrointestinal symptoms.

One possible explanation for the pain felt in IBS resides within the gradual degradation of the kynurenine pathway and serotonin depletion. Having, as a result, a pro-inflammatory cascade, these modifications of the homeostasis could, in the future, be considered as a potential biological basis in order to certify the high comorbidity between IBS and the neuropsychiatric disorders [194,195].

Unfortunately, no reliable and efficient therapy or treatment has been found for MDD-IBS patients. In several studies, the authors have tried a recovery of host’s eubiosis using probiotics [196,197], diet changes [198], being even hypothesized that in fact, the inflammasome-gut microflora system could be responsible [199,200], but only robust or negative data have been since obtained.

Data on the relevance of IBS in bipolar disorder are even more controversial. Crane et al. [201] and Mykletun et al. [202] have demonstrated that IBS is not associated with BD. On the contrary, they concluded that under certain circumstances, IBS’ severity is completely independent or slightly correlated with a reduction. The same conclusion was reached by Mykletun et al. [202] following the epidemiological study conducted on IBS women diagnosed with anxiety or depression.

Liu et al. [203] contradicted these findings following the comparison of a cohort consisting of 30,796 patients IBS patients and an identical matched control group. The authors have concluded that IBS could represent a promoter of BD. On the basis of a recent meta-analysis and nationwide studies, it has been demonstrated that, indeed, IBS could be behind the development of BD. It seems that the risk ratios primarily depend on time as the crucial variable, but the prevalence persists even after half a decade [204,205].

Analogous to MDD, Järbrink-Sehgal [206] has summarized all the available reports regarding the involvement of the gastrointestinal microflora in BD following the analysis of the 16S rRNA and one shotgun metagenomics sequencing analysis. However, no study has been identified in the current literature where probiotics were used in order to diminish BD-related symptomatology.

## 5. Conclusions

Altogether, by analyzing all the facets of pain encounters with affective disturbances, it is seen that an intricate relationship contours between them. This relationship varies from one of them being considered to be the causative factor to the other, but in the end, it is observed that this duet enhances the negative outcomes of the individual‘s state of health. In this way, it is highlighted that the presence of a clear alteration is the causative factor of distress to the person experiencing mood disorders. Therefore, the need for further researches to clarify the reasons that lay behind the numerous alterations occurring from cellular and molecular levels to clinical, psychological, and sensory levels is an obvious necessity. Also, another important aspect is to raise awareness between psychiatrists and other medical professionals to the strong connection existing between chronic pain and mood disorders, trying in this way to escape the danger of lacking a diagnostic of either chronic pain issue or mood disorder manifestation, reducing the absence of treatment, and therefore incrementing the quality of life of these patients.

## Figures and Tables

**Table 1:** Major Depressive Disorder (MDD) in pain.

Reference	Study Purpose	Diagnostic Assessment Tools	Study Description	Results	Comorbidities	Medicated or not	Quality Assessment
[42]	Rate pain, depression, and comorbid pain and depression in the context of MS (diagnosed by a medical professional)	Self-assessed; Pain: 0–10 NRS; Depression: PHQ-9; NRS was interpreted in 2 ways: any score > 0-indication of pain presence; scores ≥ 3-indicate at least moderate pain severity in persons diagnosed with MS; PHQ-9–2 ways of interpretation: variable for assessing the severity of depressive symptoms; a diagnostic tool for a major depressive episode if five or more symptoms were registered more than half the days	*N* = 161 with MS There were a majority of women (83%) Caucasian participants (97%).	The prevalence of pain and depression comorbidity with MS is only 6–19%, but when MS patients do manifest depression or pain, it is likely that the other manifestation is also present; patients suffering from pain are at least 5 times more prone to present with MDE as compared to those without pain. Also, among the individuals meeting depressive classification, 86–100% complained of pain, and 67–77% from those with depressive traits complained of at least moderate pain severity. Although individuals manifesting pain also manifested depression, the prevalence rates were lower, 11–34% met depression criteria from the ones experiencing any pain, and 15–37% from experiencing at least moderate pain.	MS	No indications	y/31
[43]	Assess chronic pain and depression in the context of MS	Self-assessed			MS		y/31
[44]	Review on increased rates of depression in chronic pain	RDC; DSM-III; DSM-III-R; DSM-IV	Reviewed in a non-structured way the literature on the connection between depression and chronic pain	Point prevalence rates of 30–54% MDD were highlighted by employing RDC and DSM criteria in diagnosing MDD in clinical samples involving chronic pain patients-indicated by 9 studies (see review). Two other studies indicated lower rates (8–21% MDD) and 2 other higher range (64–87% MDD) of MDD. 6-months and life-time MDD prevalence in 97 chronic low back pain patients indicate a 22% rate at 6-month and a 32% lifetime prevalence (Atkinson, Slater, Patterson, Grant and Garfin, 1999).	Cardiac disease: 17–18% MDD rates soon after MI; 14% MDD prevalence after 3–4 month MI; 18% MDD rates before cardiac catheterization due to suspected coronary artery disease; 27%-co-occurrence of MDD in patients scheduled for elective cardiac catheterization because of suspected coronary artery disease. Malignancy: 4–42% variability of MDD prevalence rate; Neurological conditions: 7–27% MDD rates in post-stroke patients; 22% MDD rates in Parkinson‘s disease patients. 10% MDD or another affective disorder in patients with diabetes.	No indications.	y/31
[45]	Assess chronic pain and depression influences on return to work rates	Pain: SF-MPQ; Depression: BDI;	N = 185; 84 women, 101 men; age:21–62 years; Patients could not work due to injuries: 69% back or neck injuries, 21.2% upper extremity, 0.8% lower extremity injury	Depression highly correlates with affective pain levels: r = 0.46→r = 0.43, *p* < 0.01 as compared to sensory pain rates: r = 0.36→r = 0.43, *p* < 0.01. The significant connection between the severity of depression vs. severity of pain in relation to timeframe; return to work rates were influenced by depression and pain levels.	Occupational injury	No indication.	y/31

MS = multiple sclerosis; PHQ-9 = patient health questionnaire-9; NRS= numerical rating scale; RDC = research diagnostic criteria; MI = myocardial infarction; SF-MPQ = short form McGill pain Questionnaire; BDI = Beck depression Inventory.

**Table 2:** Animal models in the context of pain and mood disorders.

Reference	Animal Model Employed	Tests Applied	Description	Results	Conclusions
**Depression in pain**					
[51]	Neuropathic pain- Chronic constriction injury (CCI) of the sciatic nerve	Electronic von Frey test (eVF): records mechanical sensitivity Elevated plus maze (EPM) test: records anxious behavior Forced swim test (FST): records depressive behavior	*N* = 64; Substance tested: tramadol 4 groups of 16 animals: sham + saline (S + S); sham + tramadol (S + T); CCI + saline (CCI + S); CCI + tramadol (CCI + T)	eVF: ↓PWT in CCI + S rats compared to S + S – hyper-nociception in CCI + S rats; ↑PWT in CCI + T rats compared to CCI + S (336%); ↑PWT in S + T rats compared to S + S (16%)—tramadol reduces mechanical sensitivity. EPM: ↓time in open arms in CCI + S vs. S + S -increased anxiety behavior in CCI + S rats; ↑time in open arms in CCI + T vs. CCI + S –tramadol reduces anxiety-behavior. FST: ↑ CCI immobility time (28%)-indicating depressive behavior; ↓ CCI + T immobility time- reduced depressive behavior; No significant effect of tramadol on sham rats.	Increased pain sensitivity in neuropathic pain animal model, along with anxious behaviour and depressive manifestations. Administration of tramadol reduces pain sensitivity, anxious behavior, and depressive manifestations.
[52]	Inflammatory pain- induction of monoarthritis (ARTH) by injecting kaolin and carrageenan into the synovial cavity of the right knee joint	Nociceptive behavior-occurrence of vocalizations at flexion-extension maneuver Pressure application measurement- limb withdrawal threshold(LWT) after noxious stimulus application Elevated plus maze(EPM): records anxious behavior Open-field test(OF): assesses locomotion ability and anxious behavior The forced swim test, Sucrose preference test: records depressive behavior following anhedonic manifestations	*N* = 44 Substance tested: amitriptyline; 2 experiments: one followed the development of mood-like disorders in ARTH rat model, second followed the amitriptyline effects on nociceptive and emotional features of the ARTH model	Experiment 1: LWT significantly decreased in ARTH rats; OF: no locomotor differences were recorded between sham and ARTH animals; ARTH rats presented with anxiety-like features; depressive-like behavior registered in ARTH animals. Experiment 2: ARTH rats treated with amitriptyline showed higher LWT compared to the saline ARTH group. Locomotor activity did not differ between ARTH with amitriptyline and ARTH without. No difference in results between ARTH with drug and without were signaled in OF or EPM; amitriptyline treated animals did not show improved depressive behavior in the FST, although ARTH rats with amitriptyline increased the climbing time but just compared to SHAM rats.	Induced experimental monoarthritis resulted in the occurrence of depressive and anxiety-like behaviors in rats. Amitriptyline administration decreased mechanical pain sensitivity but had a partial effect on depressive-like behavior.
[53]	Neuropathic pain: chronic constriction injury (CCI) of the sciatic nerve	Mechanical pain: automated algometer; Depression assessment: FST	Male Wistar rats; Substance tested: duloxetine, fluoxetine, 8-OH-DPAT	CCI vs. sham: ↓paw withdrawal thresholds; CCI + duloxetine: ↓pain sensitivity; CCI + fluoxetine: NO change; CCI-OH-DPAT: ↓pain sensitivity(from 14 ± 1 g to 25 ± 1 g, *p* < 0.001) FST: CCI vs. sham: ↑immobility time –sham +saline 115 ± 9 s vs. CCI + saline 183 ± 10 s; *p* < 0.001; CCI + duloxetine vs. CCI + saline: ↓immobility time; CCI + 8-OH-DPAT: ↓immobility time; CCI + fluoxetine: No significant changes in depression-like behaviors; CCI vs. sham: ↓climbing (sham + saline 129 ± 13 s vs. CCI + saline 62 ± 9 s); CCI + duloxetine vs. CCI + saline: No differences in climbing; CCI vs. sham: Equivalent time of swimming; CCI + duloxetine: ↑ time of swimming CCI + 8-OH-DPAT: ↑time of swimming	CCI rats presented with ↑ depressive behavior, ↑mechanical pain sensitivity. CCI + duloxetine and CCI + 8-OH-DPAT ↓depression and ↓mechanical pain, while CCI + fluoxetine had induced no significant changes.
**Pain in depression**					
[54]	Model of social defeat confrontation-induction of depressive/anxiety-like behavior.	Sweet water consumption: records anhedonic behavior-accounted for in depression; Formalin test: a model for nociception study;	There were followed the effects of the induced animal model on physiological indicators; afterward the effect of formalin in controls vs. animal model; the effects of morphine pretreatment on nociceptive formalin test, and lastly, the effects of CI-998 pretreatment or chlordiazepoxide chronic administration on the nociceptive model	Bodyweight of defeated rats was lower than of non-defeated ones (day9; 432.50 ± 7.32 g vs. 472.60 ± 6.13 g; *n* = 10; F(1,18) = 17.63; *p* < 0.001). A decrease of sucrose consumption was clearly registered in defeated rats (approx. 70% less than in undefeated ones). Five days following the conditioning sessions, increased corticosterone levels (56.59 ± 12.74 ng/mL vs. undefeated 8.84 ± 2.44 ng/mL; *n* = 10; F(1,18) = 13.41, *p* < 0.002) and adrenal glands weight in defeated rats (13.65 ± 0.25 mg/100 g vs. undefeated 9.89 ± 0.41 mg/100 g, *n* = 10, F(1,18) = 66.85, *p* < 0.0001), indicative of the increased level of stress, proof of hyperactivity of HPA axis, thus of generalized depression or anxiety manifestations. In the formalin test, defeated rats registered significantly increased pain levels in between the two phases of the nociceptive behavioral reaction and during the second phase (2.5% formalin, +205%; 6–20 min) (2.5% formalin, +47%, *n* = 113) as compared to non-defeated rats (*n* = 9). Although undefeated rats injected with formalin did not show a significant change in CCKLM cortical outflow, the defeated rats injected with formalin presented with a significant increase of dialysate CCKLM rates noted in the fifth microdialysate fraction (+46 ± 8%. t(12) = −4.3, *p* < 0.001, *n* = 13; after 2.5% formalin administration) and in the next fraction (+79 ± 10%, t(12) = −5.69, *p* < 0.0001, *n* = 13).Morphine doses on total pain rates: 2 mg/kg—a decrease of total pain scores in phase II undefeated rats(−31%) and defeated ones (−27%)	
[55]	Model of chronic depression: bilateral olfactory bulbectomy (OB)	Tail-flick test: spinal thermal pain test;	Thermal latency time was tested in naive, and either fluoxetine or morphine injected male Sprague-Dawley rats. Determinations of the autoradiographic density of specific spinal receptors (5-HT_1A_, µ-opioid) were performed	OB rats presented with increased thermal pain sensitivity compared with sham rats (*n* = 16–18, OB = 4.30 ± 0.38 s vs. *n* = 17–20, sham = 6.07 ± 0.38 s, *p* < 0.01). In the acute administration of morphine and fluoxetine, no significant differences concerning thermal pain sensitivity were recorded (*n* = 8–10 rats/group). Fluoxetine: ↑tail-flick latency until it almost matched the baseline in sham counterparts	

PWT= paw withdrawal threshold; CCI= chronic constriction injury; 8-OH-DPAT= 8-hydroxy-2[di-n-propylamino] tetralin; CCI = chronic constriction injury; FST = forced swim test.
